# Anchoring Effect of Performance Feedback on Accuracy of Metacognitive Monitoring in Preschool Children

**DOI:** 10.5964/ejop.2397

**Published:** 2021-02-26

**Authors:** Kamila Urban, Marek Urban

**Affiliations:** a Institute for Research in Social Communication, Slovak Academy of Sciences, Bratislava, Slovakia; b Department of Psychology, Faculty of Arts, Charles University, Prague, Czech Republic; c Department of History and Theory of Art, Faculty of Art and Design, Jan Evangelista Purkyne University, Usti nad Labem, Czech Republic; Kingston University, Kingston, United Kingdom

**Keywords:** preschool children, metacognitive monitoring, performance feedback, anchoring effect, predictive and postdictive judgments

## Abstract

Preschool children are generally inaccurate at evaluating past and predicting future performance. The present study examines the effect of performance feedback on the accuracy of preschoolers’ predictive judgments and tests whether performance feedback acts as an anchor for postdictive judgments. In Experiment 1, preschool children (n = 40) solved number patterns, and in Experiment 2 they solved object patterns (n = 59). The results in both experiments revealed, firstly, that children receiving performance feedback made more accurate predictive judgments and lowered their certainty after their incorrect answer. Secondly, the children relied on performance feedback more than on actual task experience when making postdictive judgments, indicating that performance feedback was used as an anchor for subsequent postdictive judgments.

Metacognitive monitoring plays an important role in human learning in areas ranging from memory and perception to reading and problem solving (Flavell, 1979; Kuhn, 2000) and it is essential in planning and evaluation of our actions (Panadero, 2017). When planning future steps, we have to predict whether we will be able to solve problems; when evaluating, we have to ask ourselves whether the steps we chose enabled us to achieve our goals (Winne & Hadwin, 1998; Zimmerman & Schunk, 2011). In other words, before we start performing a task we make predictive judgments about our future performance; and during or after the task, we make postdictive judgments about the outcomes (Schraw, 2009; Winne, 2011). When we monitor our actions accurately, our predictions and postdictions match the actual performance and we can regulate our activities effectively (Dunlosky & Rawson, 2012; Roebers & Spiess, 2017).

The ability to monitor performance in a task (Nelson & Narens, 1994) improves from preschool age (Bernard, Proust, & Clément, 2015; Destan, Hembacher, Ghetti, & Roebers, 2014; Lyons & Ghetti, 2011, 2013; Roebers, 2014; Schneider & Lockl, 2008). Preschool children already understand that their past performance should predict their future performance, but they are still overconfident in predicting performance on the new set of items (Lipko-Speed, 2013). Preschoolers are generally more accurate when making postdictions than when making predictions (Pieschl, 2009).

In order to improve the accuracy of predictive and postdictive judgments in preschool age, researchers examined the effect of performance feedback (Destan, Spiess, de Bruin, van Loon, & Roebers, 2017; Lipowski, Merriman, & Dunlosky, 2013; Urban & Urban, 2018, 2020; van Loon, Destan, Spiess, de Bruin, & Roebers, 2017). Receiving performance feedback can improve metacognitive accuracy in preschoolers’ postdictions (Urban & Urban, 2018, 2020) and predictions (Lipowski et al., 2013) from the age of 4 (Geurten & Meulemans, 2017). To answer the question of why feedback has an effect on preschool children, Geurten and Meulemans (2017) decided to test the anchoring effect of feedback on the predictive judgments of children aged 4, 6 and 8 years old in memory tasks. The anchoring effect is the adjustment of estimation based on previously presented external information, i.e., the anchor (Bahník, Englich, & Strack, 2017; Epley & Gilovich, 2006; Simmons, LeBoeuf, & Nelson, 2010; Tversky & Kahneman, 1974). They found that children adjusted their judgments in relation to feedback regardless of other accessible information related to the task. Moreover, they found a positive correlation between predisposition to adjust judgments on the basis of an anchor and inclination to rely on feedback when making memory predictions. Children who were more sensitive to the anchoring effect were also more sensitive to the feedback effect.

According to the conversational inferences theory of anchoring (Grice, 1975), the important role of social actors in the anchoring effect is emphasized; people apply implicit rules of natural conversations (such as trust related to the role of expert) in testing situation, using the anchor value to anticipate the range of possible answers. Klein et al. (2014) concluded that the anchoring effect was stronger when presented as a statement rather than a question, in participants believing the anchor had been created by a person and not a computer (Zhang & Schwarz, 2013) or when the anchor had been created by an authority rather than the participants themselves (Frederick, Mochon, & Savary, 2014). These findings can be related to Stipek and Tannatt’s (1984) assumption that children learn to accurately evaluate their performance by introjecting external feedback from important social actors. The selective accessibility theory of anchoring on the other hand explains anchoring as a combination of hypothesis-consistent testing and semantic priming (Mussweiler & Strack, 1999). In this theory, the anchor increases the accessibility of specific knowledge related to the target in the memory and the individual then tests whether the value of the knowledge in their memory matches the value of the anchor (Higgins, 1996). The anchoring effect is stronger the less knowledge people have about the target and more uncertain about the situation they are (Mussweiler & Strack, 2000; see also Bahník, Englich, & Strack, 2017).

The goal of the present study is to explore the effect of performance feedback on the accuracy of predictive judgments in preschool children and to test whether performance feedback acts as an anchor in postdictive judgments. In order to prevent possible bias occurring because of the domain specificity of metacognitive monitoring at preschool age, we designed two experiments with different kinds of tasks (number patterns in Experiment 1 and object patterns in Experiment 2).

## Overconfidence at the Preschool Age

From the developmental perspective, preschoolers are overconfident when predict their future performance (Lipko et al., 2009) and also when they judge their past performance (Destan et al., 2014; Urban & Urban, 2020). However, the overall overconfidence decreases with increasing age (O’Leary & Sloutsky, 2017; Roebers, 2002).

Preschoolers, unlike older children and adults, do not become underconfident with practice and remain overconfident over several exam sessions (Lipko et al., 2009, 2012). Underconfidence with practice is a phenomenon in which people modify previously overconfident judgments so they become underconfident after a first study-test trial (Koriat, Sheffer, & Ma’ayan, 2002). There are, however, several factors affecting overconfidence rates even at preschool age.

### Uncertainty Monitoring

The accuracy of overall monitoring depends on the ability to differentiate between correctly and incorrectly solved items (Roebers, 2014). Children can discriminate explicitly between correct and incorrect responses in perceptual tasks from the age of 3 (Lyons & Ghetti, 2011, 2013), but cannot do so in memory tasks until the age of 4 or 5 (Destan et al., 2014; Hembacher & Ghetti, 2014). That means that metacognitive monitoring in preschool age is domain specific and as such depends on the nature of the tasks. However, there is also a developmental shift. Metacognitive monitoring shifts from being domain specific to being domain general between the age of 8 to 15 years old (Geurten, Meulemans, & Lemaire, 2018; Veenman & Spaans, 2005; Vo, Li, Kornell, Pouget, & Cantlon, 2014).

Preschool children can already differentiate between correct and incorrect answers (Coughlin, Hembacher, Lyons, & Ghetti, 2015; Destan et al., 2014; Destan et al., 2017; Roebers, 2014; van Loon et al., 2017), but when they monitor incorrect answers they are still overconfident when making predictive as well as postdictive judgments (Lipko, Dunlosky, & Merriman, 2009; van Loon et al., 2017; Schneider, Visé, Lockl, & Nelson, 2000). Therefore, inaccuracy at the preschool age is most evident when children monitor incorrect answers, but at the same time, preschoolers are becoming more uncertain about their incorrect answers. Hembacher and Ghetti (2014) tested the accuracy of postdictive judgments in 3, 4 and 5 year old children. They found that 4 and 5 year old children are more certain about their correct memory answers than about their incorrect ones. Moreover, in the subsequent regulation of the next steps, children more often selected their correctly solved items to be evaluated and their incorrectly solved to be ignored. Even 3 year old children were able to select their less certain answers not to be evaluated. When children had the possibility to skip or withhold the task if they thought they do not remember the item, 3 year old children were more likely to skip a task which was not remembered (Balcomb & Gerken, 2008; Lyons & Ghetti, 2013). To summarize, children’s ability to monitor their own uncertainty develops slowly from the age of 3 (Lyons & Ghetti, 2011) and affects the regulation of next steps (Coughlin et al., 2015; Efklides, 2006; Ghetti, Hembacher, & Coughlin, 2013).

To explain the phenomenon, Pillow (2002, 2008) proposed that children could monitor uncertainty if they understood analogies (Gentner & Smith, 2012). Children are able to perform analogies if they can represent the relevant relations. Children from the age of 2 can represent binary relations and from the age of 5 to 6 they can process ternary relations (Halford & Andrews, 2014). If there is a causal relation between analogical reasoning and uncertainty monitoring, preschoolers should be able to adjust their uncertainty monitoring in relation to feedback. Previous research has supported the theory: 6 year old children still falsely believed that their incorrect answers were correct, but after receiving performance feedback their confidence in their incorrect answers fell (Destan et al., 2017; van Loon et al., 2017).

Roebers, Mayer, Steiner, Bayard, and van Loon (2019) found that 8 year old children showed an uncertainty and hesitation when monitoring incorrect answers, but they improved the detection of correctly and incorrectly solved items by gaining repeated experience with testing. Urban and Urban (2020) came to the same conclusion with high-performing preschool children. However, only high-performing preschoolers were able to foster the accuracy of their monitoring with repeated experience, low-performing preschoolers required external cues, such as performance feedback, to be able to monitor their performance more accurately. Moreover, performance feedback has been proved to be effective in improving overall monitoring accuracy in preschool children (Urban & Urban, 2018).

### Task Difficulty as a Predictor of Monitoring Accuracy

Preschoolers are more accurate when monitoring performance in easier tasks than they are in more difficult tasks (Geurten & Meulemans, 2017; Lipko et al., 2009; Roebers, 2014). Children begin to recognize task difficulty at preschool age (van Loon et al., 2017), but cannot sufficiently utilize this information when making predictions and postdictions in more cognitively demanding tasks because of working memory overload (Destan et al., 2017; Kvavilashvili & Ford, 2014; van Loon et al., 2017).

Finally, Finn and Metcalfe (2007, 2008) suggested that people are underconfident with practice because they use their initial performance in the first session as anchor for future judgments (i.e., the Memory for Past Test heuristic). Despite their performance improving in subsequent sessions they do not calibrate their judgments in relation to new information that their performance has improved, but rely instead on their initial judgment. To explain persistent overconfidence in children, Finn and Metcalfe (2014) conducted a series of experiments with children aged from 8 to 10 years old and concluded that overconfidence in children is a result of faulty memory, rather than a failure to use the anchoring effect of prior performance (see also Yang, Sun, & Shanks, 2018). This means that children are unable to remember their initial performance properly and therefore cannot calibrate their judgments more accurately.

Roebers (2014) therefore suggested that preschoolers’ monitoring judgments are linked to their ability to access a source of information or knowledge. This idea finds support in the selective accessibility theory of anchoring (Mussweiler & Strack, 1999). In other words, if children cannot access information about past performance in their memory, they cannot use that information as an anchor for future postdictive or predictive judgments; however, they could utilize an additional source of information, such as performance feedback.

## Present Study

Because of the domain specificity of metacognitive monitoring at preschool age, we still know little about children’s predictions and postdictions on solving different kind of tasks. Most research on preschoolers’ monitoring ability has used memory tasks, such as images (Destan et al., 2017; van Loon et al., 2017) or vocabulary learning (Geurten & Meulemans, 2017; Lipowski et al., 2013). In the present research, we aim to explore the effect of performance feedback using two analogical reasoning tasks. Experiment 1 focuses on numerical orders (number patterns), the symbolic arithmetic task, where children make number sequences by arranging numbers in a specific order following an example. Experiment 2 concerns patterns of objects, the non-symbolic task. The objects are arranged in a particular order and the child has to identify the pattern and then predict what follows. Numerical orders and object patterns belong to basic mathematical skills on which other mathematical concepts are based and are therefore important in later education (see Anderson & Cordes, 2013; Hawes, Nosworthy, Archibald, & Ansari, 2019; Mundy & Gilmore, 2009; Seo & Ginsburg, 2004; Xenidou-Dervou, Molenaar, Ansari, van der Schoot, & van Lieshout, 2017).

The present study has three main objectives. The first is to investigate whether preschoolers’ predictive judgments become more accurate following performance feedback and having solved several items in subsequent learning trials (Finn & Metcalfe, 2008).

The second objective concerns the effect of performance feedback on monitoring of incorrect answers. Preschool children who do not receive feedback tend not to remember incorrectly solved items (Finn & Metcalfe, 2014) and are unable to utilize their recent experience to produce more adequate judgments on subsequent performance (Lipko et al., 2009). The aim is to examine whether preschool children are able to reduce certainty about future performance (predictive judgments) after incorrectly solved items and receiving negative performance feedback.

The third objective concerns the anchoring effect of performance feedback. In research by Destan and colleagues (2017), the postdictive judgment was made following performance feedback with performance feedback having an immediate effect on the judgment. Children did not rely on their own task experience, but followed the externally administered feedback. Furthermore, van Loon et al. (2017) found that 5-year-old children did not rely on item difficulty when making judgments after receiving performance feedback. This finding was further tested by Geurten and Meulemans (2017) with similar results. Our aim is to test whether performance feedback has a stronger effect on the accuracy of postdictive judgments than task experience does. If the link between performance feedback and postdictive judgment is stronger than the link between judgment and task experience that would support the hypothesis that performance feedback acts as an anchor.

## Experiment 1

### Method

#### Participants

The sample consisted of 40 children (24 boys) aged *M* = 66.81 months (*SD* = 3.416) from 61.83 to 73.90 months. Children were recruited from state preschools in the vicinity of a university town and were native speakers. Testing was performed by a trained experimenter. Written consent was obtained from the children’s parents and verbal assent from the children.

#### Materials

The numerical order task was adapted from the mini LÜK children’s game (see Supplementary Materials, Appendix A) and served as stimuli. The children were presented with twelve items one-by-one; the answer sheet was the same for all 12 items.

The predictive and postdictive judgments were indicated on a 5-point scale. The children used a colour thermometer (Destan et al., 2017) ranging from dark blue to dark red, indicating very unsure to very sure.

#### Procedure

The trained experimenter tested the children individually in a quiet room in their preschool. Children were randomly assigned to two groups: Performance Feedback (PF); and No Feedback (NF). The detailed description of procedure can be found in Supplementary Materials (Appendix B).

The children indicated their answers by pointing to the answer sheet. After each item had been completed, the experimenter asked the child to make a postdictive judgment about his or her performance on a 5-point scale from “I am very unsure that I solved the item correctly” to “I am very sure that I solved the item correctly”.

**Figure 1:**
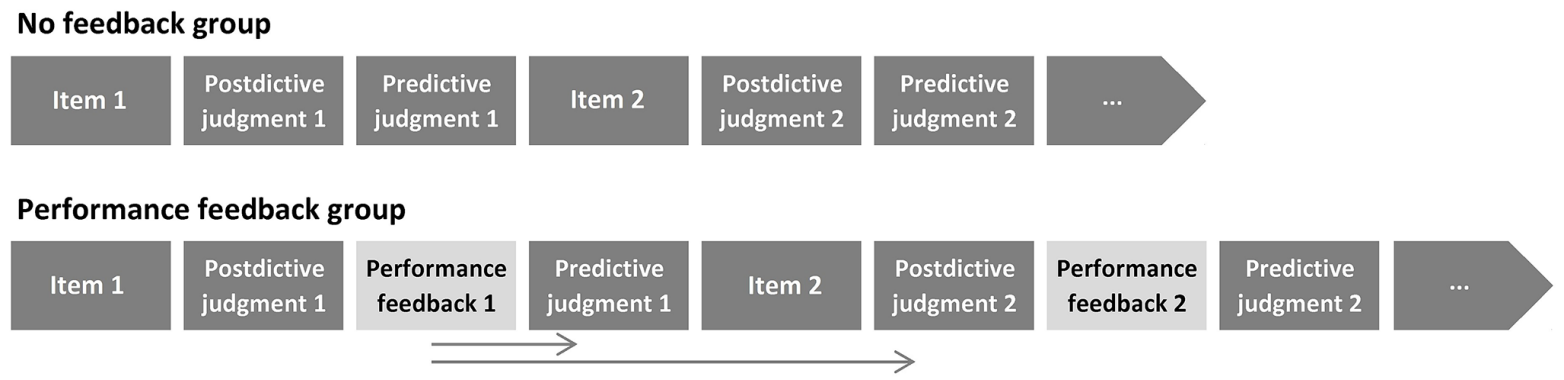
Research Procedure for NF and PF Groups *Note.* The arrows indicate the hypothesized effect of the performance feedback investigated in the research.

If the child had been assigned to the PF group then after making postdictive judgment, a child received feedback about whether the answer was correct. The experimenter then asked the child to make a predictive judgment about how he or she would perform on the next item from “I am very unsure I will solve the next item correctly” to “I am very sure I will solve the next item correctly” (see Figure 1). If the child had been assigned to the NF group, once the child had made postdictive judgment the experimenter immediately asked on the predictive judgment.

#### Analytical Approach

In the results section, three main objectives were analysed. Firstly, the general effect of performance feedback on the absolute accuracy of predictive judgments was examined. Secondly, the effect of performance feedback on the accuracy of uncertainty monitoring using predictive judgments (Model 1) was examined. Finally, analyses to explore the anchoring effect of performance feedback were conducted. To examine the anchoring effect, the postdictive judgment given directly after the item and before the performance feedback was used. Multilevel analysis in Model 2 was used to examine the effect of the performance feedback on the postdictive judgments given immediately after an incorrect answer. Another multilevel analysis in Model 3 examined the effect of performance feedback on postdictive judgments following an incorrect answer and subsequent negative performance feedback. For detailed description of analytical approach, see Supplementary Materials (Appendix C).

All the analyses were conducted in IBM SPSS (Version 25).

### Results

Descriptive statistics for the mean values of Performance, Predictive Judgments and Postdictive Judgments can be found in Table 1.

**Table 1:** Mean and Standard Deviation of Performance, Predictive Judgments and Postdictive Judgments in Experiment 1

Measure	No feedback		Performance feedback	
	*M*	*SD*	*M*	*SD*
Performance	0.31	0.47	0.35	0.48
Predictive judgments	3.69	0.99	2.98	1.23
Bias index for predictive judgments	0.63	0.23	0.40	0.34
Postdictive judgments	3.77	0.80	3.32	1.17
Bias index for postdictive judgments	0.64	0.24	0.48	0.31

*Note.* Bias Index represents underestimation (values below zero) or overestimation (values over zero) of performance. Closer to zero represents more accurate estimation.

#### Effect of Performance Feedback on Monitoring Accuracy of Predictive Judgments

To explore the effect of performance feedback on the absolute accuracy (Bias Index) of predictive judgments, a one-way ANOVA with the Feedback Group (NF, PF) as a between-subject factor was conducted. The analysis revealed a significant effect of performance feedback, *F*(1, 38) = 6.163, *p* = .018, with a large effect size $\eta_{p}^{2}$ = .14 (Cohen, 1988). The children in the PF group (*M* = 0.40, *SD* = 0.34) made more accurate predictive judgments than the children in the NF group (*M* = 0.63, *SD* = 0.23).

To examine the effect of performance feedback on predictive judgments following an incorrect answer, a multilevel analysis was conducted and Model 1 was created. Overall, the Feedback Group had a significant effect, *F*(1, 30.483) = 14.031, *p* < .001, while Item Number was non-significant, *F*(10, 42.403) = 0.968, *p* = .485, as was Interaction term, *F*(10, 42.403) = 1.364, *p* = .230. The estimates of *b*-coefficients reported a non-significant effect for Intercept (*b* = -0.227, *SE* = 0.223, *p* = .318) and a significant effect for performance feedback (*b* = 0.839, *SE* = 0.277, *p* = .006). Children in the PF group (*M* = -0.372, *SE* = 0.162) lowered their future expectations after incorrectly solved item in comparison to children in the NF group (*M* = 0.344, *SE* = 0.101).

#### Anchoring Effect of Performance Feedback on Postdictive Judgments

In Model 2 the effect of performance feedback on postdictive judgments following an incorrect answer was examined. The postdictive judgment directly followed an incorrect answer, but the performance feedback was given afterwards, so any feedback effect would be indirect only. As expected, the Feedback Group effect was non-significant, *F*(1, 150342.906) = 2.655, *p* = .103, Item Number effect was also non-significant, *F*(11, 911.089) = 1.475, *p* = .135, while the effect of Interaction term was significant, *F*(11, 911.089) = 3.000, *p* = .001. The estimates of *b*-coefficients reported a non-significant effect for Intercept (*b* = -0.140, *SE* = 0.251, *p* = .579) and a non-significant effect for performance feedback (*b* = 0.664, *SE* = 0.345, *p* = .054). Children in the PF group (*M* = -0.170, *SE* = 0.191) did not monitor their incorrect answers significantly more accurate than children in the NF group (*M* = 0.260, *SE* = 0.182).

The aim of Model 3 was to assess the effect of performance feedback on postdictive judgments made following an incorrect answer and negative performance feedback. Previous Model 2 showed that performance feedback had a non-significant effect on uncertainty monitoring when the judgment did not immediately follow the performance feedback. On the contrary, in Model 3, performance feedback had a significant effect, *F*(1, 25.473) = 8.894, *p* = .006, Item Number did not, *F*(10, 177.857) = 0.615, *p* = .800, nor did the Interaction term, *F*(10, 177.857) = 1.590, *p* = .113. The estimates of *b*-coefficients reported a non-significant effect for Intercept (*b* = -0.406, *SE* = 0.313, *p* = .197) and a significant effect for performance feedback (*b* = 0.883, *SE* = 0.400, *p* = .028). Children in the PF group (*M* = -0.262, *SE* = 0.166) made significantly lower postdictive judgments following an incorrect answer and negative feedback in comparison to children in the NF group (*M* = 0.287, *SE* = 0.080).

Moreover, a comparison of the parameters for the Feedback Group in Model 2 (*b* = 0.664, *SE* = 0.345, *p* = .054) and in Model 3 (*b* = 0.883, *SE* = 0.400, *p* = .028) indicated that performance feedback had a stronger influence on subsequent postdictive judgments regardless of actual task experience. This supported the hypothesis that performance feedback acts as an anchor.

### Discussion on Experiment 1

Preschool age children are often inaccurate when judging their learning performance. When making predictions about future performance, preschoolers do not become underconfident with practice and are overconfident across several trials (Lipko et al., 2009). The current study supports these findings: the preschoolers who received no feedback did not become more accurate in their predictions across items. However, children who received performance feedback became more accurate in their subsequent predictions (Lipowski et al., 2013). Moreover, children in the feedback group lowered their future expectations after they had received performance feedback and were less sure in their subsequent judgment. Our research supports the previous conclusion that children usually predict they will get the answer right, but if they receive negative information about their performance (negative performance feedback following an incorrect answer), they are less certain than children who do not receive feedback (Kvavilashvili & Ford, 2014).

Moreover, preschool age children can use the external information about their performance as an anchor not just for the next prediction but also for subsequent postdictive judgments. They utilized the performance feedback from the previous item more than they utilized their own task experience in the subsequent item. This finding is in line with Finn and Metcalfe (2007, 2008) who showed that children rely on the explicit information given by the experimenter and not on their previous performance. In addition to Geurten and Meulemans (2017) who studied the anchoring effect of feedback on predictive judgments, results of the current study support the anchoring effect of performance feedback on postdictive judgments of preschool children.

Task difficulty could affect the accuracy of metacognitive monitoring at this age. Preschool children seem to find it difficult to monitor more cognitively demanding tasks (Destan et al., 2017; Kvavilashvili & Ford, 2014). In the case of difficult tasks children from the age of 4 use a perceived task difficulty as a guide in their judgments instead of actual task experience (Geurten, Willems, Germain, & Meulemans, 2015). We therefore conducted Experiment 2 based on object pattern tasks. Object patterns are also part of basic math skills but different to the number patterns in nature (Resnik, 2000; see also Dehaene et al., 1998). Ability to solve a nonsymbolic task (Experiment 2) does not depend on the ability required to perform the symbolic arithmetic task (Experiment 1). Solving the arithmetic task depends on understanding of abstract representations of numbers, which tends to develop from school age, whereas nonsymbolic knowledge develops earlier, at preschool age, influencing the ability of preschoolers to accurately monitor performance in these tasks (Barth, La Mont, Lipton, & Spelke, 2005; Rubinsten, Henik, Berger, & Shahar-Shalev, 2002).

## Experiment 2

### Method

#### Participants

The sample consisted of 59 children (31 boys) aged *M* = 68.86 months (*SD* = 6.011) from 41.80 to 79.80 months. All the children were recruited from state preschools in the vicinity of a university town and were native speakers. A trained experimenter performed the testing. Written consent was obtained from the children’s parents and verbal assent from the children.

#### Materials

The children completed 12 analogical reasoning items, adapted from the children's mini-LUK game, in the same order. The children had to identify the pattern of objects and then predict what followed (see Supplementary Materials, Appendix D).

#### Procedure

See Supplementary Materials (Appendix B).

#### Analytical Approach

See Supplementary Materials (Appendix C).

### Results

Descriptive statistics for the mean values of Performance, Predictive Judgments and Postdictive Judgments can be found in Table 2.

**Table 2:** Mean and Standard Deviation of Performance, Predictive Judgments and Postdictive Judgments in Experiment 2

Measure	No feedback		Performance feedback	
	*M*	*SD*	*M*	*SD*
Performance	0.34	0.48	0.47	0.50
Predictive judgments	3.23	1.00	3.09	1.07
Bias index for predictive judgments	0.47	0.23	0.30	0.20
Postdictive judgments	3.45	0.89	3.34	0.90
Bias index for postdictive judgments	0.53	0.17	0.36	0.19

*Note.* Bias Index represents underestimation (values below zero) or overestimation (values over zero) of performance. Closer to zero represents more accurate estimation.

#### Effect of Performance Feedback on Monitoring Accuracy of Predictive Judgments

To explore the effect of performance feedback on the absolute accuracy (Bias Index) of predictive judgments, a one-way ANOVA with the Feedback Group (NF, PF) as a between-subject factor was conducted. The analysis revealed a significant effect of performance feedback, *F*(1, 57) = 9.176, *p* = .004, with a large effect size $\eta_{p}^{2}$ = .139. The children in the PF group (*M* = 0.30, *SD* = 0.20) made more accurate predictive judgments than the children in the NF group (*M* = 0.47, *SD* = 0.23).

To examine the effect of performance feedback on predictive judgments following an incorrect answer, a multilevel analysis was conducted and Model 1 was created. Overall, performance feedback had a significant effect, *F*(1, 58.811) = 6.348, *p* = .014, while Item Number was non-significant, *F*(10, 280.114) = 1.062, *p* = .392, as was the Interaction term, *F*(10, 280.114) = 1.762, *p* = .067. The estimates of *b*-coefficients reported a non-significant effect for Intercept (*b* = -0.265, *SE* = 0.246, *p* = .282) and a significant effect for performance feedback (*b* = 1.031, *SE* = 0.319, *p* = .001). Children in the PF group (*M* = -0.213, *SE* = 0.130) lowered their future expectations after incorrectly solved item in comparison to the children in the NF group (*M* = 0.227, *SE* = 0.117).

#### Anchoring Effect of Performance Feedback Using Postdictive Judgments

Model 2 examined the effect of performance feedback on postdictive judgments made immediately after an incorrect answer. As we expected, the effect of the Feedback Group was non-significant, *F*(1, 68.059) = 0.144, *p* = .706. The effects of Item Number, *F*(11, 51.007) = 2.410, *p* = .017, as well as Interaction term, *F*(11, 51.007) = 3.151, *p* = .003, were significant. The estimates of *b*-coefficients reported a non-significant effect for Intercept (*b* = 0.171, *SE* = 0.196, *p* = .388) and a non-significant effect for performance feedback (*b* = 0.190, *SE* = 0.255, *p* = .461). Children in the PF group (*M* = -0.018, *SE* = 0.093) did not monitor their incorrect answers significantly more accurate in comparison to children in the NF group (*M* = 0.032, *SE* = 0.091).

Model 3 concerned the effect of performance feedback on the second postdictive judgment given after an incorrect answer. The analysis revealed that the Feedback Group had a significant effect, *F*(1, 54.430) = 6.821, *p* = .012, Item Number did not, *F*(10, 57.042) = 1.275, *p* = .266, nor did Interaction term, *F*(10, 57.042) = 1.614, *p* = .126. The estimates of *b*-coefficients reported a non-significant effect for Intercept (*b* = -0.165, *SE* = 0.195, *p* = .402) and a significant effect for performance feedback (*b* = 0.729, *SE* = 0.242, *p* = .005). Children in the PF group (*M* = -0.227, *SE* = 0.128) showed significantly lower postdictive judgments following an incorrect answer and negative feedback in comparison to the children in the NF group (*M* = 0.184, *SE* = 0.092).

The results indicate that preschool children ameliorated their postdictive judgments in line with the performance feedback regardless of an actual task experience. Comparing parameters of Model 2 (*b* = 0.171, *SE* = 0.196, *p* = .388) and Model 3 (*b* = 0.729, *SE* = 0.242, *p* = .005), this supported the hypothesis that performance feedback has an anchoring effect.

### Discussion on Experiment 2

In Experiment 2 the children solved reasoning task involving patterns of objects. Similarly as in Experiment 1 the preschool children made significantly more accurate predictions about their future performance after receiving performance feedback (Geurten & Meulemans, 2017). Performance feedback also had an effect on uncertainty monitoring, with children who received feedback after an incorrect answer lowering their future expectations. Overall improvement in monitoring accuracy following feedback provides further evidence of the importance of external cues in monitoring development at preschool age (Urban & Urban, 2018, 2020).

Similarly as in Experiment 1, the examination of the postdictive judgments in Experiment 2 provided further support that performance feedback acts as an anchor. The performance feedback effect was transferred to the judgment regardless of task experience. In other words, children relied on the external information rather than internal cues (van Loon et al., 2017).

Moreover, the children that received no feedback in Experiment 2 were able to utilize the internal cues from task experience; however, once they had received performance feedback, they relied on an external cue. This result can be related to the nature of the task since object pattern task employed in Experiment 2 is developmentally more appropriate than the number pattern task used in Experiment 1 (Barth et al., 2005). This result can also provide further support for the conclusion that preschool children find highly cognitively demanding tasks more difficult to monitor (Destan et al., 2017; Kvavilashvili & Ford, 2014) and that it is important to maintain a level of task difficulty that is developmentally adequate so the children remain motivated to proceed (Geurten et al., 2015; Schneider, 1998; Shin, Bjorklund, & Beck, 2007).

## General Discussion

The first aim of this study was to examine monitoring accuracy of predictive judgments in preschool children. In the two experiments preschool children solved analogical reasoning tasks based on patterns. Experiment 1 contained number pattern task and Experiment 2 contained object pattern task. In the pattern based task the children had to systematically explore how the various elements were repeated and find the number or object that followed. Patterning is an important part of basic mathematical skills that is important in the early years of school age (Anderson & Cordes, 2013; Hawes et al., 2019; Seo & Ginsburg, 2004). The object patterns are nonsymbolic in nature, while the number patterns are symbolic and the ability to solve them depends on the child understanding abstract representations of numbers, which develops from school age (Barth et al., 2005; Mundy & Gilmore, 2009).

### Effect of Performance Feedback on Monitoring Accuracy of Predictive Judgments

From the age of 4, children understand that their past performance predicts their future performance and therefore children's second predictions for the same set of tasks are more accurate than the firsts. Moreover, children use their past performance as a cue for subsequent predictions. However, they have no valid cues for making predictions for new set of tasks (Lipko-Speed, 2013). For this reason, in order to foster the accuracy of predictive judgments, Geurten and Meulemans (2017) decided to provide an external cue to the preschoolers. They adapted calibration feedback consisting of information about performance as well as about the monitoring accuracy. However, young children that are not yet able to process ternary relations (Halford & Andrews, 2014) or the low-performers (Urban & Urban, 2018, 2020) may have difficulties to take into account this rather complex external cue, but they are able to improve their monitoring accuracy when performance feedback is provided.

In both experiments conducted in this study, the effect of performance feedback on the overall accuracy of predictive judgments was large ($\eta_{p}^{2}$ = .140 and $\eta_{p}^{2}$ = .139). The finding that preschool children ameliorated their predictions in line with feedback is consistent with previous research findings (Lipowski et al., 2013). More importantly, in both experiments the preschoolers were able to utilize performance feedback to reduce certainty about future performance after incorrectly solving an item. The ability to monitor uncertainty is important for subsequent regulation of actions, for example modifying learning strategy, like skipping an item or seeking help and plays an important role in self-regulation of learning in primary education (Coughlin et al., 2015; Destan et al., 2017; van Loon et al., 2017).

### Anchoring Effect of Performance Feedback Using Postdictive Judgments

The second aim of the study was to test whether performance feedback acts as an anchor on postdictive judgments. The anchoring effect is the adjustment of estimation based on previously presented external information, an anchor (Bahník, Englich, & Strack, 2017). Geurten and Meulemans (2017) conducted a series of experiments and found that children who were more sensitive to feedback effect were also more sensitive to the anchoring effect. More importantly, they concluded that feedback acts as an anchor on predictive judgments regarding memory tasks, because the children in their research relied on performance feedback rather than on task difficulty. This result is in line with research by van Loon and colleagues (2017) who studied the utilization of internal and external cues in making postdictive judgments in memory tasks. They found preschoolers who received no feedback used task difficulty as an internal cue when making postdictive judgments; however, when feedback was received, the external information became more important than task experience.

Both the experiments conducted in this study support these conclusions: the preschool children making postdictive judgments relied more on performance feedback than on task experience and thus, by definition (Tversky & Kahneman, 1974), performance feedback acted as an external cue serving as an anchor for the children’s judgments. The cue utilization hypothesis (Koriat, 1997) assumes that there is always an interplay between several internal, external or mnemonic cues which accounts for the accuracy of judgments. Roebers et al. (2019) stated that cue utilization is a factor driving recognition improvements over time. However, this theory cannot explain why an individual making judgments decides to rely on one particular cue and not another (Koriat, 2016; Winne, 2011).

Therefore to understand why the preschoolers chose to use the externally administered performance feedback instead of task experience we can turn to theories related to the anchoring effect, such as conversational inferences theory (Grice, 1975). This theory states that people making judgments transfer social rules from everyday life (e.g., trust in the expertise of scientists or superior knowledge of teachers) to the test situation, and so they tend to believe that the feedback (anchor) administered by the experimenter is informed and close to the actual result (Jacowitz & Kahneman, 1995). This theory has been criticised because anchoring effect also occurs in situations where feedback is extremely unlikely (e.g., when using number 212 as an anchor for the age of Mahatma Gandhi; see Bahník, Englich, & Strack, 2017). However, this is not the case with performance feedback in our designs because participants could trust that the experimenter’s feedback was correct and could be relied upon when making subsequent judgments. Raaijmakers, Baars, Schaap, Paas, and van Gog (2017) in a similar setting manipulated performance feedback in order to test the effect of dominantly positive or dominantly negative feedback and none of the participants noticed them doing so. Future research should address whether the perceived validity of judgments relies on social rules by explicitly questioning the validity of the experimenter or feedback. This could examine whether participants rely on feedback even when compromised, administered by an “uninformed” experimenter lacking credibility or that is inconsistent with the participants’ internal cues (see also Baadte & Kurenbach, 2017; Brunot, Huguet, & Monteil, 1999).

Furthermore, the selective accessibility theory of anchoring (Mussweiler & Strack, 1999) states that anchor improves accessibility of topic related knowledge in the memory. Individuals then test whether knowledge in their memory matches the value of the anchor. This is resulting in stronger anchoring effect in unfamiliar situations, where the individual lacks expertise or feels uncertain (Mussweiler & Strack, 2000). Research by Schneider (1998) shows that children’s judgments have higher metacognitive accuracy when they are doing familiar tasks, because they can relate to their previous experience. However, in the research on feedback effect there are often used unfamiliar tasks to ensure the results are unbiased (Geurten & Meulemans, 2017; Urban & Urban, 2019; van Loon et al., 2017). As Winne (2011) pointed out students perform better on unfamiliar tasks when the overall evaluation standards are set externally by the teachers (Hamilton, 1985; Kiewra, Benton, Kim, Risch, & Christensen, 1995), but once students gain experience of the subject they can begin to use task familiarity as an internal cue on their own (Begg, Anas, & Farinacci, 1992). However, the high degree of familiarity with the ideas prevents the feedback from having an effect, which is especially alarming when related to prejudice or stereotype reduction (Swire, Ecker, & Lewandowsky, 2017). Applying this theory to preschoolers, future research should address differences in the effect size of performance feedback in familiar and unfamiliar tasks to see whether as preschoolers gain more experience of the task, the performance feedback loses its’ potential to improve accuracy in metacognitive monitoring.

Both conversational inferences theory and selective accessibility theory of anchoring have therefore practical implications in administration of feedback in educational process. Stipek and Tannatt (1984) found that children from preschool age, perceiving their teachers as authorities, introject teachers’ feedback and use it as the general assessment criteria for their own success. The repetition of information without any feedback can, on the other side, results in the reverse situation when children introject the information itself. Plichtová (2013) described the situation in which students were unable to accept feedback on the familiar concepts and an attempt to administer feedback was perceived as a personal threat. Moreover, Urban (2017) in research with art students described that individuals become reluctant to pursue personal goals when receiving negative feedback from peers or teachers. These results are in line with research on performance feedback in which Venables and Fairclough (2009) concluded that feedback following failure led to adverse changes in mood and motivation and participants were on the verge of abandoning the task. Winne (1996) therefore proposed that it is necessary to create learning environment allowing individuals to decide when to adjust knowledge in line with external feedback and when to rely on their own internal cues, such as personal experience or problem familiarity. In this context, the conceptual connection between anchoring effect theory and feedback administration may bring us closer to understanding the cue selection process that begins at preschool age.

## Notes

For this article the following Supplementary Materials are available (Urban & Urban, 2021):

- Figures showing tasks used in Experiments 1 and 2.
- Detailed description of procedure.
- Analytical Approach.
